# Benthic‐pelagic coupling drives non‐seasonal zooplankton blooms and restructures energy flows in shallow tropical lakes

**DOI:** 10.1002/lno.10241

**Published:** 2016-03-18

**Authors:** Alfred Burian, Michael Schagerl, Andrew Yasindi, Gabriel Singer, Mary Nakabungo Kaggwa, Monika Winder

**Affiliations:** ^1^ Department of EcologyEnvironment and Plant Sciences, Stockholm UniversityStockholmSweden; ^2^ Department of Limnology and Bio‐OceanographyUniversity of ViennaViennaAustria; ^3^ Department of Biological SciencesEgerton UniversityNjoroKenya; ^4^ Department of EcohydrologyLeibniz‐Institute of Freshwater Ecology and Inland Fisheries (IGB)BerlinGermany; ^5^ Department of Wildlife and Aquatic Resources ManagementUniversity of RwandaHuyeRwanda

## Abstract

Zooplankton blooms are a frequent phenomenon in tropical systems. However, drivers of bloom formation and the contribution of emerging resting eggs are largely unexplored. We investigated the dynamics and the triggers of rotifer blooms in African soda‐lakes and assessed their impact on other trophic levels. A meta‐analysis of rotifer peak densities including abundances of up to 6 × 10^5^ individuals L^−1^ demonstrated that rotifer bloom formation was uncoupled from the food environment and the seasonality of climatic conditions. A time series with weekly sampling intervals from Lake Nakuru (Kenya) revealed that intrinsic growth factors (food quality and the physicochemical environment) significantly affected rotifer population fluctuations, but were of minor importance for bloom formation. Instead, rotifer bloom formation was linked to sediment resuspension, a prerequisite for hatching of resting‐eggs. Population growth rates exceed pelagic birth rates and simulations of rotifer dynamics confirmed the quantitative importance of rotifer emergence from the sediment egg‐bank and signifying a decoupling of bloom formation from pelagic reproduction. Rotifer blooms led to a top‐down control of small‐sized algae and facilitated a switch to more grazing‐resistant, filamentous cyanobacteria. This shift in phytoplankton composition cascaded up the food chain and triggered the return of filter‐feeding flamingos. Calculations of consequent changes in the lake's energy budget and export of aquatic primary production to terrestrial ecosystems demonstrated the large potential impact of nonseasonal disturbances on the functioning of shallow tropical lakes.

## Introduction

In temperate aquatic ecosystems, a major driver of variations in phytoplankton and zooplankton abundances is the seasonal interplay between bottom‐up and top‐down controlling factors (Sommer et al. 2012). Contrastingly, organisms in tropical systems have been perceived to live in an “endless summer” lacking large seasonal fluctuations in temperature and irradiance. The relatively constant environmental conditions were originally hypothesized to result in small temporal variations in the density and age structure of tropical plankton populations (Twombly 1983). Comparative investigations of temperate and tropical population dynamics have demonstrated a significant increase of intra‐annual variation in primary production rates with latitude (Melack 1979), but did not reveal systematic differences in annual variation of phytoplankton biomass (Kalff and Watson 1986). Consequently, the seasonality and the drivers of observed temporal fluctuations in the tropics became a focal point of research (Melack 1988; Masundire 1994; Ka et al. 2011).

Pronounced seasonal patterns have been detected in many deep tropical lakes (Gliwicz 1986; Masundire 1994). While variations in irradiance and temperature play only a minor role, rainfall and wind patterns influence nutrient and suspended sediment concentrations and constitute the main seasonal drivers determining phytoplankton growth rates (Talling 1986; Ndebele‐Murisa et al. 2010). Zooplankton often follows phytoplankton peaks with a lagged response (Dumont et al. 1994) and seems to be mainly indirectly influenced by seasonal cues. Further, also non‐cyclic drivers affect plankton population dynamics in tropical lakes (Vareschi and Jacobs 1985) and are contribute to large interannual differences (Gliwicz 1986; Dumont et al. 1994). Possible drivers of non‐cyclic dynamics are extreme weather events (Robarts et al. 1998), nonlinear or chaotic biological interactions in rapidly overturning plankton communities (Beninca et al. 2008), and the mass‐emergence of resting stages from sediment egg‐banks (Gliwicz 1986; Masundire 1994). The hatching of zooplankton resting stages is mainly coupled to changes in light, salinity, temperature, or oxygen concentrations (Pourriot and Snell 1983) and occurs after a dormancy period of variable length (Gilbert and Schroder 2004). In the tropics, the lack of large fluctuations of temperature and irradiance may turn the disturbance or oxygenation of the sediment surface into a critical trigger for the emergence of zooplankton resting eggs.

Shallow tropical lakes are, in contrast to deep lakes, characterized by strong benthic‐pelagic coupling causing naturally enriched nutrient levels (Oduor and Schagerl 2007b). Closed‐basin soda‐lakes, a frequent lake type in East‐Africa well known for its large flocks of lesser flamingos, show continuously high nutrient concentrations due to the lack of river out‐flows, which leads to elimination of nutrients as a major seasonal driver. Zooplankton in tropical soda‐lakes is dominated by small‐sized species and as in many tropical systems (Fernando 2002; Fernandez et al. 2012) large‐bodied cladocerans and calanoid copepods are rare. Instead, rotifers can play an important ecological role (Vareschi and Jacobs 1985), reaching average densities of 10³ individuals L^−1^ to 10^4^ individuals L^−1^ and forming dense blooms of over 10^5^ individuals L^−1^ (Iltis and Riou‐Duwat 1971; Vareschi and Vareschi 1984; Burian et al. 2014). Peak biomass values belong to the highest recorded densities of metazoan zooplankton world‐wide (Jeppesen et al. 2000; Auer et al. 2004) and are likely to impact the food‐web structure and energy budgets of tropical soda‐lakes. Besides their ecological importance, rotifer blooms are commonly formed within few days and represent well‐definable events, which provide a suitable opportunity to study drivers, timing and periodicity of zooplankton blooms in tropical systems.

Here, we compiled the annual dynamics of rotifers and potential environmental drivers from seven African soda‐lakes monitored over a cumulative time‐span of 230 months, and analysed the seasonality of rotifer bloom‐occurrence. Further, a weekly time series of environmental and biological variables from Lake Nakuru (Kenya) allowed us to investigate drivers of rotifer blooms. In a partial redundancy analysis (pRDA), we distinguished between intrinsic and extrinsic drivers of growths and thereby determined the effect of physicochemical conditions, the food environment and resting‐egg hatching on rotifer population dynamics. We compared the results with simulations of rotifer population growth, which helped us to identify triggers of rotifer blooms and pinpoint differences and similarities in bloom formation between tropical and temperate lake ecosystems.

## Materials and methods

### Data compilation for the meta‐analysis

We included datasets from soda lakes that covered a minimum sampling period of 6 consecutive months sampled at monthly or shorter intervals. Altogether, 413 samples from seven lakes (ephemeral and perennial systems, see Supporting Information Table S1 and S2) around Lake Chad (Iltis and Riou‐Duwat 1971) and along the eastern branch of the African Rift valley (Vareschi and Vareschi 1984) were considered, including data from L. Bogoria and L. Nakuru collected within this study (weekly sampling in both lakes for a period of 14 months, sampling as described below for L. Nakuru). All lakes shared several limnological characteristics: primary producers are typically dominated by filamentous cyanobacteria (mainly by *Arthrospira fusiformis* and several *Anabaenopsis* species; Krienitz et al. 2013), but shift at times to pico‐ and nanophytoplankton communities. The most abundant zooplankton species in all lakes were the rotifers *Brachionus plicatilis* Mueller, *Brachionus dimidiatus* Bryce and *Hexarthra jenkinae* Beauchamp (Vareschi and Jacobs 1984). Besides rotifers, lesser flamingos (*Phoeniconaias minor* Geoffroy Saint‐Hilaire) are typically present and a major consumer in all lakes (Vareschi and Jacobs 1985). The fish fauna in soda‐lakes is restricted to the occasional occurrence of soda‐tilapia (*Oreochromis alcalicus* Hilgendorf).

### Timing of rotifer blooms

To investigate the cyclicity of zooplankton blooms, rotifer densities from all lakes were transformed into a binomial variable, where 1 represents the onset of a bloom and 0 all other states of rotifer dynamics. The onset of a bloom was defined by (1) a population increase of > 200% between sampling intervals and (2) rotifer biomass exceeding 4 g dry mass m^−3^ or the upper limit of a lake‐specific 90% confidence interval of mean biomass. Lake‐specific 90% confidence intervals for mean biomass were incorporated in the definition of bloom onsets to account for varying productivity among individual lakes. Environmental variables influencing the probability of rotifer bloom formation were identified using a mixed‐effect logit‐regression model with salinity, chlorophyll *a* (as a proxy for resource availability) and two measures of seasonality as fixed‐effect predictor variables. Lake identity was included as a random effect to account for random variation among lakes. We used two different measures of seasonality, the average monthly rainfall (mm/m^3^) and the days since the start of the rainy season; calendar days are not an appropriate measure of seasonality. Environmental variables were Z‐standardized across all lakes before analysis (see Supporting Information Tables S1, S2 for lake‐specific data). In a second mixed‐effect logit‐regression, we analysed the impact of food quality on rotifer bloom formation. We used a subset of the total dataset (*n* = 229; seven lakes) for which phytoplankton data were available and evaluated the importance of the relative biovolume of five phytoplankton groups (filamentous cyanobacteria, single‐celled cyanobacteria, cryptophytes, chlorophytes, and a diatom‐chrysophyte group) on the probability of rotifers to form blooms. Regression models with all possible combinations of explanatory variables were created and the most parsimonious predictive models were selected based on the Akaike Information Criterion in all analyses (AIC; Burnham and Anderson 2002). No temporal autocorrelation was found in time series of dependent variables.

### Rotifer population dynamics in Lake Nakuru

A time series from L. Nakuru was used to investigate drivers of rotifer blooms and associated life‐history parameters. Data were collected weekly for six months (January 2009 to July 2009) at a central off‐shore station. Water samples were collected from the surface using a Schindler trap (10 L). The sampler integrated water from the first meter in the water column, representing approximately the euphotic zone of L. Nakuru. Measurements included water temperature, specific conductivity, pH (all measured with WTW multiprobe 340i, Weilheim, Germany), Secchi‐depth, dissolved organic carbon (DOC; TOC‐VCPH analyser; Shimadzu, Kyoto, Japan), nutrient concentrations (total dissolved nitrogen and soluble reactive phosphorus; modified spectrophotometric standard procedures), suspended particulate matter (PM), and Chl *a* (cold acetone extraction). In addition, we quantified the biomass of heterotrophic bacteria, phytoplankton, heterotrophic flagellates, ciliates, rotifers, mesozooplankton, and flamingos (for detailed method description see supporting information, Appendix S1). We calculated in situ growth rates (*r*) of rotifer population based on

(1) $$r=(\text{ln}\;N_{2}-\text{ln}\;N_{1})/(t_{2}-t_{1})$$

where *N*_2_ and *N*_1_ represent rotifer densities at time *t*_2_ and *t*_1_. Birth rates (*b*) were calculated from egg development times (*D_E_*), densities of female rotifers (*N*_♀_) and densities of rotifer eggs attached to females (*N_E_*) after Vareschi and Jacobs (1984).

(2) $$b=\text{ln}\;(1\;+\;N_{E}*D_{E}{}^{-1}*N_{♀}{}^{-1})$$

Death rates (*d*) were obtained by subtracting *r* from *b*.

During onsets of rotifer blooms, we compared changes in in situ populations with simulations of rotifer densities computed with fixed *b* and *d*. Whereas *b* was based on counts of pelagic egg, simulations for different *d* were created, ranging from negative values (mimicking recruitment of resting eggs from the seed‐bank, Andrew and Fitzsimons 1992; Gilbert and Schroder 2004) and *d* values incorporating age losses (*d* = 0.11, based on average life span measurements of 9.5 d; Pourriot and Rougier 1975) to mean in situ mortalities (*d* = 0.43, based on field measurements of Vareschi and Jacobs 1984 from L. Nakuru). Details about the parameterisation of egg development time and calculation of corrected egg‐ratios (*N_E_* × $N_{♀}^{-1}$) used in the simulations are given in the supporting information, Appendix S2.

Partial redundancy analysis (pRDA, Borcard et al. 1992) was used to test whether environmental variables explained biomass fluctuations of the three dominating rotifer species, *B. plicatilis*, *B. dimidiatus*, and *H. jenkinae*. We separated environmental variables that could intrinsically drive changes in population densities from those that affect resting eggs hatching and external contributions to pelagic populations. Intrinsic drivers of growth included temperature, salinity, Secchi depth and five resource parameters [Chl *a*, biomass of nano‐ and picoplankton, small ciliates (< 60 *μ*m), and the filamentous algae *Anabaenopsis* spp. and *A. fusiformis*], which are known to affect rotifer growth and reproduction in L. Nakuru (Burian et al. 2013; Burian et al. 2014). Large ciliates were not included because they are outside the prey size‐range of rotifers. Secchi depth reflected underwater light conditions and was included as an indicator of optical prey‐detection by soda‐tilapia and the predation‐vulnerability of rotifers. Although data from L. Nakuru are not available, we assumed soda‐tilapia to be the main predator of rotifers because zooplankton is an important part of its diet in L. Magadi (pers. comm. Rodi Ojoo). The relative importance of different intrinsic drivers of growth was tested in a multiple regression using AIC for model selection. Residuals were normally distributed and no temporal autocorrelation was found.

The PM concentration was used as an explanatory variable driving hatching from resting eggs. PM was included because oxygen and light conditions were only near the water surface of L. Nakuru suitable for stimulating resting egg hatching, suggesting sediment resuspension to be a likely prerequisite for rotifer resting‐egg hatching. PM was a good proxy for sediment resuspension because total plankton biomass contributed little to PM (< 20% throughout the sampling period). We used total PM instead of inorganic PM because large proportions of the sediments in L. Nakuru consist of faeces and detritus with a high organic content and inorganic PM is influenced by salinity and precipitation of dissolved salts. Secchi depth was only weakly correlated to PM (*r* ^2^ = 0.17, *p* = 0.03) because of the influence of dissolved organic matter on the under‐water light climate. This allowed the inclusion of both Secchi depth and PM as predictors in the pRDA. We repeated the pRDA analysis with offsets of 0–7 weeks between species and environmental datasets to investigate a possible delay in the response of the rotifer community. We kept the size of the dataset (*n* = 28) constant by using weekly rotifer data from July 2009 to August 2009. Bonferroni corrections of *p*‐values were applied to account for the repetition of the statistical analysis.

To detect shifts in the phytoplankton community structure, we tested whether break‐points were detectable in the time series of algae < 40 *μ*m and of algae > 40 *μ*m. We used the penalized contrast method of Lavielle to identify the number of break‐points in phytoplankton time series (Lavielle 2005). All statistical tests were performed using the open source software R, version 3.1.1 (R Development Core Team 2014).

## Results

### Meta‐analysis of rotifer blooms

The full dataset encompassed 413 sampling occasions with 36 blooms, resulting in an average bloom frequency of one bloom every 5.2 months. The highest recorded rotifer biomass was 52 g DM m^−3^ (Table 1), which is equivalent to around 6.2 × 10^5^ individuals L^−1^. The rotifers *H. jenkinae, B. dimidiatus*, and *B. plicatilis* were found in all study lakes, with the two *Brachionus* species contributing over 90% to the total rotifer biomass in more than 95% of the samples.

**Table 1:** Mean, standard deviation (SD) and maximum (Max) rotifer densities of Lake Nakuru (Jan–Jul 2009), ephemeral African soda‐lakes (yearly desiccation) and all seven African soda‐lakes included in the meta‐analysis. Densities are presented in g dry mass m^−3^.

	L. Nakuru (Jan–Jul 2009)			Ephemeral lakes			African soda‐lakes		
Species	Mean±SD (%)		Max	Mean±SD (%)		Max	Mean±SD (%)		Max
*Brachionus dimidiatus*	2.22	±140	13.14	4.05	±175	33.62	0.96	±308	33.62
*Brachionus plicatilis*	2.73	±126	13.21	1.76	±214	18.56	1.10	±340	24.01
*Hexarthra jenkinae*	0.40	±181	2.84	0.31	±243	3.57	0.10	±389	3.57
Total	5.36	±103	17.99	6.11	±167	52.18	2.16	±257	52.18

**Figure 1:**
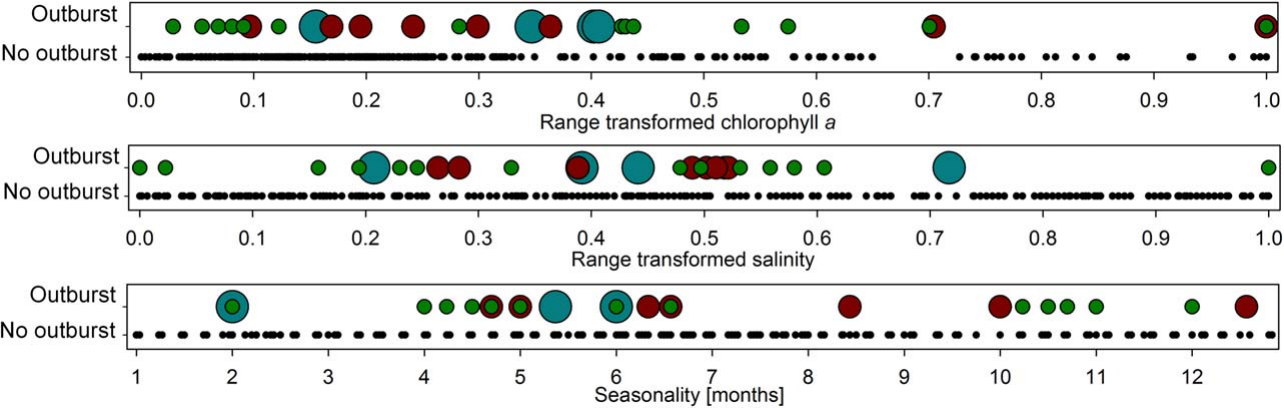
Emergence of rotifer blooms (rotifer biomass > 4 g DM m^−3^ and doubling of biomass since last sampling) in relation to the range of Chl *a* (top), salinity (center) and seasonality (bottom) in seven African soda‐lakes: Every circle represents one sampling event, colored circles represent onset of rotifer blooms (small, green circles 4–8 g DM m^−3^; medium, red circles 8–20 g DM m^−3^; large, blue circles ≥ 20 g DM m^−3^). Seasonality was normalized to the beginning of the main rainy season. Chl *a* and salinity were range‐transformed to values between 0 and 1 to facilitate an analysis of blooming probability across lakes.

Rotifer bloom events were distributed across the full range of salinity (5.1 mg L^−1^ to 62.5 mg L^−1^; Supporting Information Fig. S1) and Chl *a* levels (11 *μ*g L^−1^ to 1650 *μ*g L^−1^), and across all seasons, with no perceptible clustering of blooms (Fig. 1). A logit‐regression analysis revealed that both, Chl *a* (*z*‐value = 0.79; *p* = 0.43) and seasonality (defined by start of rainy season: *z*‐value = −0.30; *p* = 0.77; average monthly rainfall: *z*‐value = −0.74; *p* = 0.46), were not significantly related to rotifer blooming probability within single lakes. A negative effect on bloom probability was found for salinity (*z*‐value = −2.78; *p* > 0.01), but high deviance indicated limited goodness of fit of the model, i.e., wide scattering of data relative to model predictions. In a second logit‐regression we tested the influence of relative biovolume of different algae groups on rotifer bloom formation. None of the algae groups had a significant impact on rotifer bloom occurrence (*p* > 0.1), highlighting a decoupling between phytoplankton community composition and the onset of rotifer blooms.

### Triggers of rotifer blooms in Lake Nakuru

**Figure 2:**
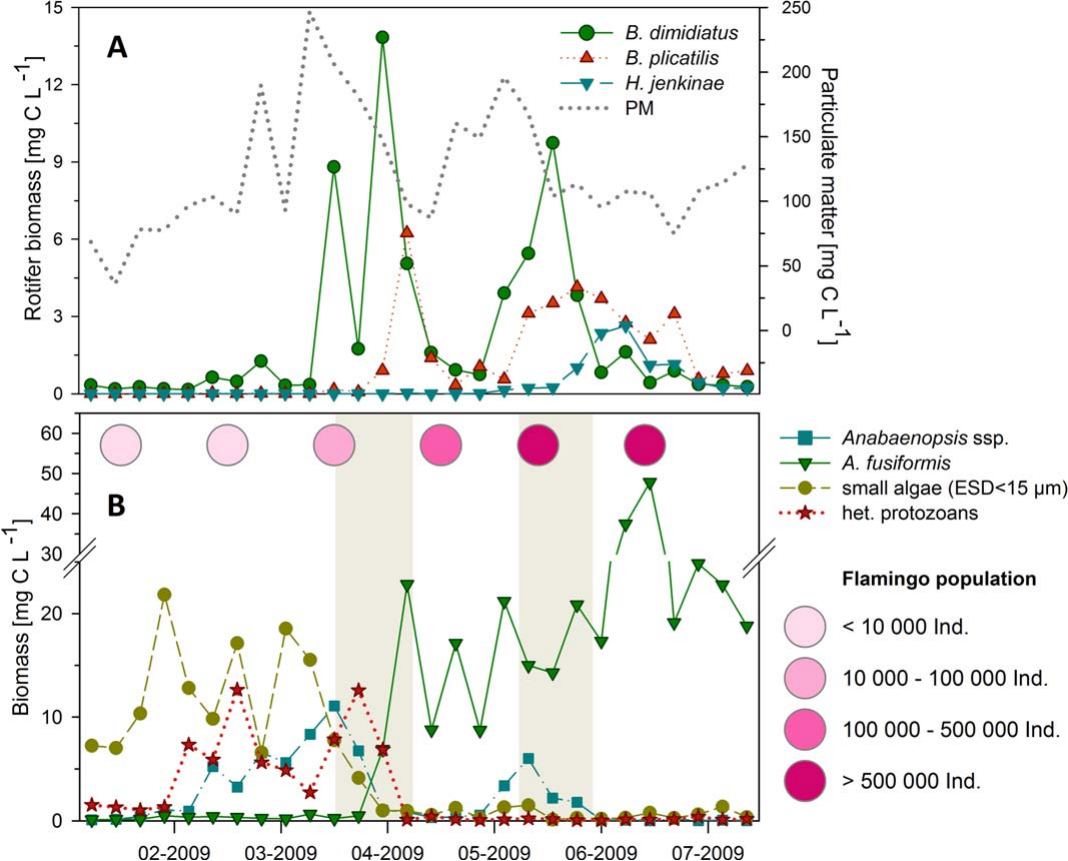
Dynamics of the most abundant rotifer species and particulate matter (A) and of available food particles (B) in Lake Nakuru from January to July 2009. In (B), periods of rotifer blooms and monthly flamingo densities are represented by grey bars and pink circles, respectively. PM = particulate matter; ESD = equivalent spherical diameter.

In accordance with the results of the meta‐analysis, our investigation of the population dynamics of algae, heterotrophic protozoans and rotifers in L. Nakuru showed that the emergence of rotifers was not tied to distinct prey communities. While the onset of a first rotifer bloom occurred in the presence of dense populations of nano‐, microalgae and small omnivorous ciliates, a second rotifer bloom four weeks later developed during dominance of the spirally‐coiled cyanobacterium *Arthropsira fusiformis* (> 20 mg C L^−1^, > 95% phytoplankton biomass, Fig. 2) and higher densities of large herbivorous ciliates.

We investigated the dataset of the meta‐analysis to examine impacts of rotifer blooms on algae community composition. The relative contribution of filamentous cyanobacteria significantly increased when densities at onsets of rotifer blooms were compared to prebloom densities (paired *t*‐test, *p* = 0.28; see Fig. S2). An analysis of phytoplankton time series in L. Nakuru revealed one break‐point each in the datasets of small algae (< 40 *μ*m) and of filamentous cyanobacteria. While small algae decreased strongly in sampling week 11, filamentous cyanobacteria showed a swift increase two weeks later (Fig. 2B). The break‐points of both phytoplankton communities were co‐occurring with the first rotifer bloom (weeks 11–13), demonstrating that rotifer bloom formation can be tied to large changes in phytoplankton communities. The reestablishment of filamentous cyanobacteria populations was concurrent with the return of flamingos to the lake (Fig. 2B), which use *A. fusiformis* as their main food source.

**Figure 3:**
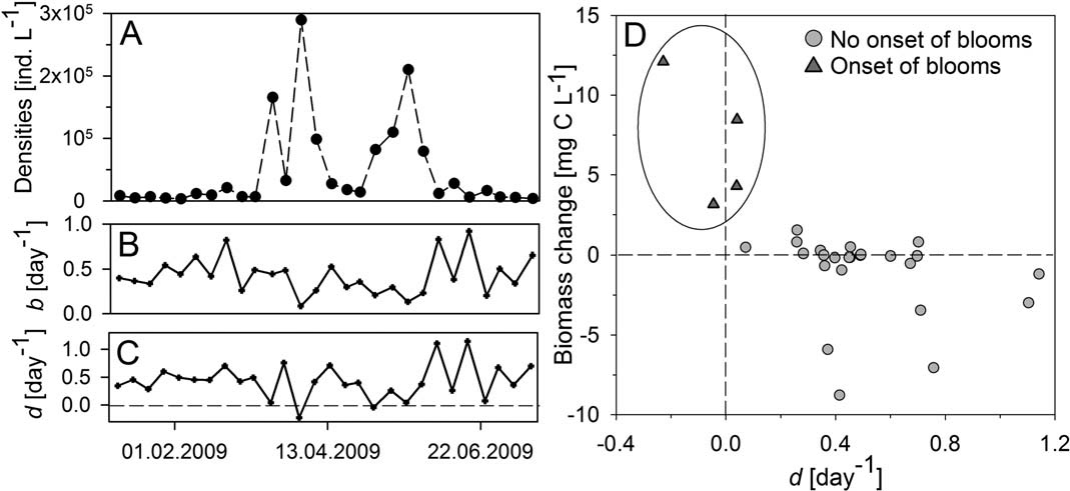
Changes of *B. dimidiatus* (A) population densities, (B) birth and death rates and (C) relationships between death rates and weekly absolute changes in biomass between January and July 2009 in Lake Nakuru. The circle highlights all sampling dates with a population change rate of > 5000 individuals d^−1^, which were marked by distinctly different death rates.

The pRDA analysis revealed that rotifer dynamics were significantly influenced in similar magnitude by intrinsic drivers of population growth and by hatching of resting eggs (Fig. 3). A multiple regression investigating the relative importance of different intrinsic predictor variables revealed that temperature and the biomass of pico and nanoplankton were the most important intrinsic drivers. Both were included in the regression‐model with the lowest AIC. An immediate response of rotifer communities to variables affecting intrinsic growth was observed (*r* ^2^ = 0.37; adjusted *p* < 0.05). In contrast, a three week time‐lag (*r* ^2^ = 0.44; adjusted *p* < 0.01) between peaks of rotifer biomass and PM, which served as proxy for resting egg hatching, suggested different response times of rotifer populations to different environmental drivers.

**Figure 4:**
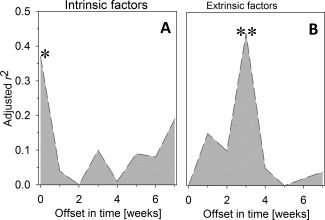
Goodness of fit for pRDA using two environmental data sets to explain rotifer dynamics in Lake Nakuru. The analysis was repeated with an offset of 0–7 weeks between species and both environmental datasets. (A) Results for variables potentially affecting intrinsic population growth rates (adjusted for effects of particulate matter), (B) results for particulate matter as a proxy of rotifer resting‐egg hatching (adjusted for factors affecting intrinsic growth). Significant models are displayed as * (*p* < 0.05) and ** (*p* < 0.01).

Onsets of rotifer blooms were not synchronised with high rotifer birth rates and egg production (Fig. 4). In contrast, rotifer death rates were indicative of massive rotifer resting egg hatching at the onset of bloom periods: *d* of both *Brachionus* species were close or even below 0 d^−1^ whenever a major increase of rotifer abundance occurred. While the average *d* of *B. dimidatus* was 0.43 d^−1^, death rates < 0.05 d^−1^ during onsets of blooms were significantly different from *d* during other sampling days (two‐sample *t*‐test, *p* < 0.001; Fig. 4C). Negative death rates signify that birth rates were too low to account for observed population growth rates, suggesting that external recruitment via resting‐egg hatching supported outbreaks of rotifer biomass.

**Figure 5:**
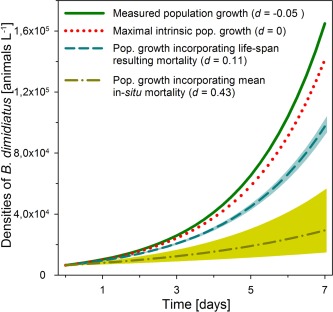
Measured and simulated population growth curves of *B. dimidiatus* during the onset of the first rotifer bloom in Lake Nakuru (09 March 2013–17 March 2013). Three different scenarios were modelled based on a death rate *d* of 0 (maximal intrinsic population growth), a death rate of 0.11 resulting from age losses and a death rate of 0.43, the mean death rate of field populations in Lake Nakuru. Lightly coloured areas represent 95% confidence intervals.

Simulations of rotifer population growth rates based on field measurements of the first *B. dimidiatus* bloom in L. Nakuru (Fig. 5) indicated a large quantitative effect of external recruitment on rotifer bloom formation. Model outputs yielded in densities of 1.0 × 10^5^ individuals L^−1^ after a 7 d growth period, when death rates included age‐related mortality but no predation (*d* = 0.11 d^−1^). The modelled population growth was much lower than monitored field densities of 1.65 × 10^5^ individuals L^−1^. After including predation‐based in situ death rates (Vareschi and Jacobs 1984), the modelled rotifer density even decreased to values between 1.5 and 5.5 × 10^4^ individuals L^−1^. Hence, a conservative calculation based on the upper level of the 95% confidence level of a model incorporating predatory losses predicts that the emergence of resting eggs and the reproductive success of these newly hatched individuals accounted for two thirds of the observed population growth or 10^5^ individuals L^−1^ within 7 d.

## Discussion

Plankton blooms are essential features of marine and freshwater habitats, affecting productivity and energy transfer between trophic levels. Our study of rotifer dynamics in African soda‐lakes revealed the three following major findings: (1) rotifer blooms are frequent, noncyclic and non‐seasonal events that emerge independently of food quantity and quality, (2) resuspension of benthic resting eggs can play a major role in formation of pelagic rotifer blooms, (3) rotifer blooms can be accompanied by marked transitions in prey communities. The resulting changes can cascade up the food‐web potentially leading to marked transitions in food‐web structure and even in the energy and nutrient budgets of shallow tropical lakes.

### Triggers of rotifer blooms

Our results show that food quantity and quality were not major drivers of rotifer bloom formation. Tropical soda‐lakes belong to the most productive systems world‐wide, reaching gross photosynthesis rates of up to 30 g O_2_ m^−2^ d^−1^ (Talling and Wood 1973; Melack and Kilham 1974; Melack 1981). Although communities of filamentous cyanobacteria and single‐celled algae alternate (Vareschi 1982; Ballot et al. 2004; Schagerl and Oduor 2008), food particles for zooplankton are in surplus most of the time (Iltis and Riou‐Duwat 1971; Melack 1988). Food quality, represented by the taxonomic composition of prey, was likewise not a trigger of rotifer blooms, despite earlier studies showing selective foraging behaviour of rotifers in tropical soda‐lakes (Burian et al. 2013, 2014). Rotifer blooms occurred in times of dominance by highly nutritional microalgae as well as by low food‐quality cyanobacteria (lacking sterols and highly unsataurated fatty acids, Martin‐Creuzburg et al. 2008; Sperfeld and Wacker 2012). Thus, our results demonstrate an uncoupling of rotifer bloom formation from food resources in tropical soda‐lakes.

The pRDA analysis demonstrated that intrinsic and extrinsic growth factors had significant effects of similar importance on rotifer population dynamics in L. Nakuru. Intrinsic growth factors, however, seemed to affect mainly weekly population fluctuations and not rotifer bloom formation. Bottom‐up and top‐down controlling factors were uncoupled from onset of blooms because no substantial increase in egg ratios and no large decreases in predator densities (e.g. fish kills or flamingo migrations) were observed at times of bloom formation. In contrast, peaks of PM concentration, indicating sediment resuspension and the initiation of resting‐egg hatching, was strongly correlated with onsets of rotifer blooms (Fig. 2A; Supporting Information Fig. S4), turning the sediment egg‐bank into a potential driver of bloom formation.

Resting‐egg hatching was confirmed as a key trigger of bloom formation by an offset of population growth rates and pelagic birth rates, mirrored by low or even negative death rates during onsets of blooms. Accordingly, our simulations of rotifer population growth demonstrated the quantitative importance of resting‐egg hatching for pelagic population dynamics. Although negative death rates are commonly interpreted as strong evidence for resting egg hatching in field populations (Andrew and Fitzsimons 1992), an exact quantification of benthic emergence is difficult. In our dataset it is impossible to differentiate between rotifers hatching from resting eggs and the off‐spring they produce until the next sampling date, leading to a potential overestimation of external recruitment. Nonetheless, high rotifer densities, similar to those monitored during onsets of blooms, are known to trigger the formation of new resting eggs (Serra et al. 2005). A negative correlation between *B. dimidiatus* densities and birth rates in L. Nakuru (*r* ^2^ = 0.17; *p* = 0.03) indicates that increased sexual reproduction could have led to a potential underestimation of resting egg hatching by the number of sexually produced resting eggs entering the sediment egg‐bank. In spite of these uncertainties, our population models show the quantitative importance of emerging resting eggs; even conservative calculations predict more than half of the population during onsets of blooms to originate from benthic egg banks.

External recruitment and resulting rotifer blooms also influenced the structure of pico‐ and nanophytoplankton communities. In an analysis of data across soda‐lakes, we observed a significant increase in the relative abundance of filamentous cyanobacteria during onsets of rotifer blooms. The weekly sampling in L. Nakuru revealed that single‐celled algae decreased quickly after the onset of rotifer blooms. Community grazing rates of > 700 mg C L^−1^ d^−1^ (based on average indivudal ingestion rates on small algae populations, Burian et al. 2013) were about 10 times higher than the average daily gross primary production (Oduor and Schagerl 2007a). Interestingly, despite being a potential food source for rotifers (Ka et al. 2012; Burian et al. 2014), the filamentous cyanobacterium *A. fusiformis* was not negatively affected by rotifers. This is in agreement with findings suggesting cyanophages as driving the decline of *A. fusiformis* blooms (Peduzzi et al. 2014). Instead, one rotifer bloom in L. Nakuru seemed to facilitate the transition from single‐celled algae to *A. fusiformis*, whose stability can likely be attributed to a higher grazing resistance. Previous field studies revealed that *A. fusiformis* can adapt its colony shape in response to high grazer densities (Kaggwa et al. 2013), that rotifers select for single‐celled species in controlled grazing experiments (Burian et al. 2013) and that *A. fusiformis* is ingested in situ only by the larger‐sized *B. plicatilis* and not by *B. dimidiatus* (Burian et al. 2014). Thus, *A. fusiformis* seems to be able to compensate grazing losses and even profit from rotifer blooms as they lead to the elimination of competitors, provide an alternative food for planktivorous fish and increase dissolved nutrient levels through recycling and sloppy feeding (Supporting Information Fig. S3).

The rotifer‐driven switch of phytoplankton communities also triggered a shift in the food‐web structure of L. Nakuru and changed energy flows within the system. Protozoan communities switched from small (< 60 *μ*m) bacterivorous and omnivorous ciliates (mainly *Euplotes* sp. and *Cyclidium* sp.), to the large filamentous‐cyanobacteria‐feeding genus *Frontonia*, which—in contrast to smaller species—is outside the prey‐size spectrum of rotifers (Burian et al. 2013). Moreover, the change in primary producers improved the habitat suitability of L. Nakuru for lesser flamingos. A bloom of filamentous cyanobacteria does not necessarily attract large flocks of flamingos because flamingo migration also depends on food conditions in other African soda‐lakes, but it does create the ecological opportunity for high flamingo densities (Krienitz and Kotut 2010). In L. Nakuru, the flamingo population increased from < 10,000 to > 500,000 individuals within six weeks after the reestablishment of *A. fusiformis* (Fig. 2B). If present in high abundances, lesser flamingos are the dominant herbivores in African soda‐lakes (Vareschi and Jacobs 1985). Populations of > 500,000 individuals ingest > 60% of daily primary production (Vareschi 1979). The decline of bacterivorous ciliates and the return of flamingos, therefore, impacted energy flows in L. Nakuru: only one third of the biomass ingested by flamingos returns in form of faeces and dead flamingos to the lake (Vareschi 1979), whereas the other two thirds are metabolically utilised (assimilated and respired) or transported to terrestrial ecosystems (faeces and urine dropped over land and predation loss to terrestrial predators). Consequently, short non‐cyclic sediment disturbances can trigger pelagic dynamics that cause an ecosystem‐shift from rapid internal energy‐cycling driven by microbial organisms to a state where approximately 40% of daily primary production is removed from aquatic food webs.

### Patterns of rotifer emergence

Hatching of zooplankton resting stages in temperate ecosystems affects biodiversity, species co‐existence and the speed of evolution (Caceres 1999; Hairston and Kearns 2002; Brendonck and De Meester 2003). Field hatching rates, however, are relatively low and negative death rates have been observed only during low population densities (Arndt 1988; Andrew and Fitzsimons 1992; Hairston et al. 2000). We showed that in tropical soda‐lakes resting‐egg hatching can also be of major quantitative importance reflecting mechanistic differences to other lake types.

The pattern of rotifer emergence in L. Nakuru was characterised by (1) highly synchronised resting egg hatching within species, (2) a temporal succession with *B. plicatilis* following the smaller‐sized *B. dimidiatus*, and (3) a time‐lag between environmental triggers and the hatching of resting eggs. Latent periods between stimulus and the actual hatching are well known from laboratory experiments (Gilbert and Schroder 2004) and range from a few hours to several months (Duggan et al. 2002). To date, it remains unclear whether these temporal patterns of emergence are primarily genetically predefined or environmentally triggered.

In *Artemia salina* (Van Der Linden et al. 1986) and probably also in *B. plicatilis* (Hagiwara et al. 1995), light and reactive oxygen species lead to the degradation of the pigment haematin, which impedes the hatching of resting stages (Vanvlasselaer and De Meester 2010). The haematin concentration in resting eggs influences the lag‐time between stimulation and hatching and is genetically predefined. Still, the mode of exposure to environmental cues needs to be considered. Although during our study‐period the pRDA revealed a significant time‐lag of three weeks, the delay in hatching seemed to be variable. A broader exploration of sampling data from L. Nakuru (weekly rotifer data from three stations were available for 14 months, but excluded from statistical analyses because of missing phytoplankton data) disclosed that the time‐lag between stimulation and hatching varied between 0 weeks and 6 weeks within the same population (Supporting Information Fig. S4). This illustrates that initial haematin levels and the mode of exposure influences the timing of resting egg hatching. The synchronised hatching and the consistent temporal succession of rotifer species, however, are probably genetically encoded and the result of an unknown, but strong selection pressure.

### Plankton blooms in temperate and tropical habitats

**Figure 6:**
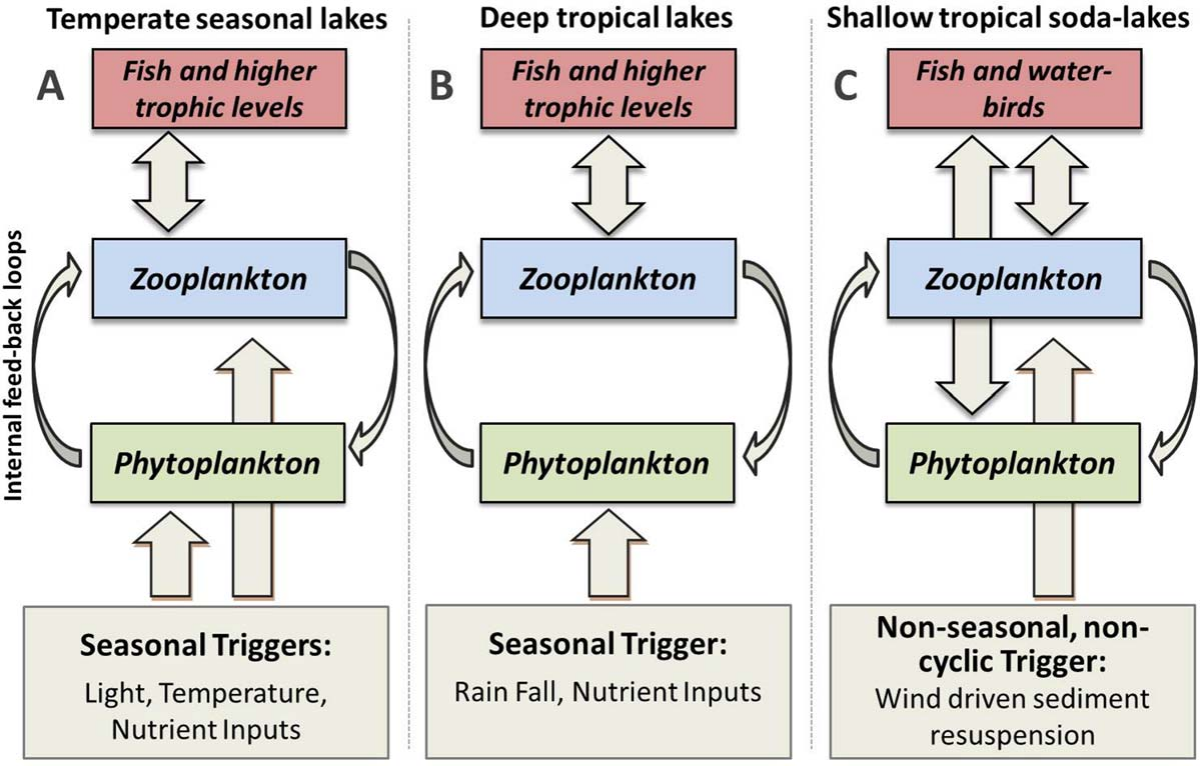
Triggering of zooplankton blooms in (A) temperate seasonal (B) tropical seasonal and (C) tropical soda‐lakes. Onset of zooplankton blooms in tropical soda‐lakes are decoupled from food resources and triggered directly by non‐cyclic factors. Phytoplankton, which is only weakly influenced by seasonal cues (Talling 1986), constitutes an important food‐source for fish and water‐birds in tropical soda‐lakes.

Whereas plankton dynamics in deep temperate lakes are governed by changes in irradiance, temperature, nutrient availability and resulting biological interactions (Sommer et al. 2012), the relative importance of temperature and irradiance is lower in the tropics (Ndebele‐Murisa et al. 2010). The direct influence of seasonal triggers on zooplankton is therefore reduced in deep tropical lakes, and seasonal cycles of zooplankton dynamics are dependent on indirect channelling of seasonal influences via phytoplankton growth cycles (Fig. 6). The shallowness of tropical soda‐lakes, however, may lead to a surplus of dissolved nutrients and thus decouples phytoplankton dynamics from seasonal changes in environmental conditions, causing non‐seasonal patterns to dominate in planktonic food‐webs.

Nevertheless, environmental factors play an important role in tropical soda‐lakes. Our study demonstrates that zooplankton blooms are triggered by resting egg hatching and are probably related to wind‐driven resuspension of sediments. Moreover, African soda‐lakes show a large degree of interannual variability in rainfall and evaporation (Vareschi 1982). Consecutive changes in lake levels and salinity can likewise lead to changes in species dominance and community composition and support the high variability of plankton communities (Schagerl et al. 2015).

Despite differences in plankton seasonality between African soda‐lakes and other lake types, several characteristics of plankton blooms are shared across habitats (Fig. 6). A common feature in all systems is that biological feed‐backs emerge when algae with low grazing resistance dominate during the onset of zooplankton blooms. In such cases, high community consumption rates during blooms first trigger the overexploitation of food resources, then a density‐dependent collapse of zooplankton populations, and finally the establishment of a more grazing resistant algae community. In tropical soda‐lakes, however, zooplankton blooms emerge independently of the composition of phytoplankton communities, sometimes muting internal feed‐backs. Generally, similar processes, such as mixing, nutrient supply rates, and consumer‐resource interactions drive plankton dynamics in temperate and tropical systems, but their relative roles and ultimate driving factors (temperature, irradiance, precipitation, and wind patterns) vary with the shallowness and latitudinal position of lake‐types.

## Supporting information

Supporting Information
