# Supplementary material for: Benthic‐pelagic coupling drives non‐seasonal zooplankton blooms and restructures energy flows in shallow tropical lakes
Source: Limnol Oceanogr. 2016 Mar 18;61(3):795–805. doi: 10.1002/lno.10241 (PMC4981947; doi:10.1002/lno.10241)
Supplement: Supplementary file 1 — Supporting Information [file LNO-61-795-s001.docx]

**Electronic Supplementary Information**

**Table S1** List of data sets included in the meta-analysis of the impact of external factors on the blooming-probability of rotifers in African soda-lakes. Lakes in Chad were situated between 13°14 ̍ N -14°07 ̍ N and 14°10 ̍ E - 15°04 ̍ E, longitude and latitude of sampling points in L. Nakuru and L. Bogoria were 00°21 ̍ S 36°06 ̍ E and 00°16 ̍ N, 36°05 ̍ E respectively.

| **Lake** | **Country** | **Lake type** | **Length of data set [d]** | **sampling interval** | **Year** | **Reference** |
| --- | --- | --- | --- | --- | --- | --- |
| 4th Lake of Mombolo | Chad | perennial | 549 | monthly | 1967-68 | (Iltis & Riou-Duwat, 1971) |
| Bodou | Chad | perennial | 549 | monthly | 1967-68 |  |
| Bogoria | Kenya | perennial | 484 | weekly | 2008-09 | This study |
| Latir | Chad | ephemeral | 1280 | monthly | 1964-68 | (Iltis & Riou-Duwat, 1971) |
| Maou | Chad | ephemeral | 305 | monthly | 1967-68 |  |
| Nakuru | Kenya | perennial | 1658 | monthly | 1972-76 | (Vareschi & Jacobs, 1985) |
|  |  |  | 483 | weekly | 2008-09 | This study |
| Ouadi de Liwa | Chad | perennial | 457 | monthly | 1967-68 | (Iltis & Riou-Duwat, 1971) |
| Rombou | Chad | perennial | 580 | monthly | 1967-68 |  |

**Table S2** Limnological characteristics of lakes included in the meta-analysis investigating the impact of external factors on the blooming-probability of rotifers in African soda-lakes. Blooming probability is calculated as blooms per month.

|  | Chl *a* [µg L^-1^] | | Salinity [mg L^-1^] | | Max. lake level [m] | | SO4 [meq L^-1^] | | CO3 [meq L-1] | | HCO3 [meq L-1] | | Blooming probailitiy |
| --- | --- | --- | --- | --- | --- | --- | --- | --- | --- | --- | --- | --- | --- |
|  | **Mean ± SD** | | **Mean ± SD** | | **Max** | **Mean ± SD** | | | **Mean ± SD** | | **Mean ± SD** | | **Mean** |
| 4^th^ Lake of Mombolo | 239.6 | ±59 | 5.6 | ±0.4 | 1.9 | 28.1 | | ±4.4 | 424.4 | ±43 | 147.3 | ±25 | 0.13 |
| Bodou | 827.1 | ±146 | 25.9 | ±2.8 | 1.5 | 28.1 | | ±4.4 | 424.4 | ±43 | 147.3 | ±25 | 0.07 |
| Bogoria | 317.0 | ±164 | 42.9 | ±3.7 | 7.5 | 0.7 | | ±0.4 | 415.4 | ±45 | 575.5 | ±66 | 0.20 |
| Latir | 282.8 | ±206 | 31.2 | ±17.6 | 0.4 | 1372.0 | | ±202 | 1526.5 | ±561 | 226.5 | ±179 | 0.30 |
| Maou | 295.4 | ±183 | 31.8 | ±16.6 | 0.5 | 111.8 | | ±NA | 118.0 | ±NA | 113.0 | ±NA | 0.08 |
| Nakuru | 454.3 | ±282 | 25.9 | ±11.7 | 1.5 | 1.8 | | ±1.0 | 250.7 | ±141 | 338.3 | ±182 | 0.08 |
| Ouadi de Liwa | 263.3 | ±165 | 16.0 | ±4.2 | 0.3 | 13.8 | | ±10.6 | 340.1 | ±155 | 86.7 | ±48 | 0.33 |
| Rombou | 177.8 | ±128 | 14.5 | ±2.1 | 1.0 | 21.8 | | ±4.3 | 181.6 | ±53 | 70.7 | ±22 | 0.31 |

**S1:** *Sampling of Lake Nakuru and Lake Bogoria*

The two saline-alkaline Kenyan Rift Valley lakes Bogoria and Nakuru were sampled at weekly time intervals for 14 months between 07/2008 and 10/2009. Both lakes were sampled at three off-shore stations (north, south and centre). The full environmental data set, including ciliate, flagellate and phytoplankton counts were only available for a central stations and a core period.

Rotifer samples were concentrated using a 50 µm sieve (smaller mesh sizes led to clogging of filamentous algae) and fixed with 5% formalin. Individuals and eggs were counted under an inverted microscope at 200x magnification. Biovolume of the various taxa were estimated using geometric approximations of the various body and cell shapes (Sun & Liu, 2003). Group-specific conversion factors for the biovolume-biomass conversion were used (Table S3). Bacterial samples were fixed (5% formalin), stained with SYBR Gold (Tuma et al., 1998) and counted under a compound epifluorescence microscope (Motic BA 400, Nikon, Tokyo). Phytoplankton samples were fixed (5% formalin) and counted using an inverted microscope (Nikon Diaphot, Nikon, Tokyo) according to Utermöhl (1958). Two magnifications, one for pico- and nanoplankton (1000x) and one for larger taxa (200x), were used. Protozoan samples were fixed with Bouin’s solution (5%) and stained using the Quantitative Protargol Staining Technique (QPS) by Montagnes & Lynn (1993) to facilitate counting under a compound-microscope.Flamingo populations were estimated in monthly intervals by visual inspection from the shore-line in a drive from the north to the south end of the lake.

Lake Bogoria is split into three distinct basins. Freshwater inflow and wind exposure, which affects the accumulation of cyanobacteria blooms on the water surface, differ strongly among basins. For that reason and as rotifer population dynamics at the different stations seemed to be independent of each other, we included data from the three basins as separate data sets in the meta-analysis. Visual inspection of the rotifer dynamics of L. Nakuru collected at the different sampling stations disclosed a connection between different stations and therefore only data from the central station were used in the meta-analysis.

**Table S3** Conversion factors used to calculate C (carbon) content of various biota.

| **Taxa** | **Conversion factors** | **Reference** |
| --- | --- | --- |
| Heterotrophic bacteria | C content per cell [fg cell^-1^] = 0.025 | Bell ([1993](#_ENREF_2)) |
| Cyanobacteria | C content per biovolume [pg µm^-3^] = 0.22 | Ahlgren ([1983](#_ENREF_1)) |
| Protist plankton | log C [pg cell^-1^] = log -0.665 + 0.939 x log V [µm³] | Menden-Deuer and  Lessard ([2000](#_ENREF_3)) |
| Bacillariophyceae | log C [pg cell^-1^] = -0.610 + 0.892 x (log plasma V [µm^3^]) | Strathmann ([1967](#_ENREF_5)) |
| Rotifers | % C [dry mass] = 7.8 x V [10^6^ µm]^-0.37^ | After Telesh et al. ([1998](#_ENREF_6)) |

**S2:** *Parameterization of egg development time and correction of egg ratios*

To calculate birth rates we used egg development times of 0.91 for *B. dimidiatus* and 1.01 for *B. plicatilis*, which are both values measured in L. Nakuru ([Vareschi and Jacobs 1984](#_ENREF_7)). For *Hexarthra* *jenkinae* we used an egg development time of 0.6, measured in ephemeral desert ponds at 20 °C ([Schroder et al. 2007](#_ENREF_4)).

For the simulation of maximum intrinsic rotifer population growth rates, we used corrected egg-ratios. It is not possible to differentiate in fixed samples between juvenile females, who have an average development time of about 1 day ([Vareschi and Jacobs 1984](#_ENREF_7)), and non-egg-carrying adult females. For the calculation of birth rates, however, the number of “adult females” is required. We, therefore, attempted to overcome this short-coming of the counting data by the application of the following correction:

We based our calculations on the assumption that the growth conditions stayed constant from *t*_-1_ until *t*_7_, where *t*_0_ represented the sampling on the 24.03.2009 and *t*_7_ the sampling on the 31.03.2009. We then established a circular system of equations consisting of the following five steps: (i) The number of adult females (*N*_♀_) at *t*_0_ is equal to the number of female rotifers (*N*_R_) at *t*_0,_ minus the number of juvenile female rotifers (*N*_J_) at *t*_0_. (ii) *N*_J_ at *t*_0_ can be calculated by multiplying the egg ratio at *t*_0_ with the number of *N*_R_ at time *t*_-1_. (iii) *N*_R_ at time *t*_-1_ can be calculated from equation 1 (see methods) based on *N*_R_ and the population growth rate *r*. (iv) *r* is equal to the birth rate *b* minus the death rate *d*. We used a conservative estimate of 0.3 for *d* (average for *B. dimidiatus* during the sampling period was 0.43). As *r* was therefore *b* – 0.3 and (v) as *b* is dependent on *N*_♀_, (see methods, equation 2) the circular equation system was closed. We then split this circular equation system into two sets of equations and found one common solution for both sets of equations with an iterative calculation approach, leading to a corrected egg-ratio for time *t*_0_.


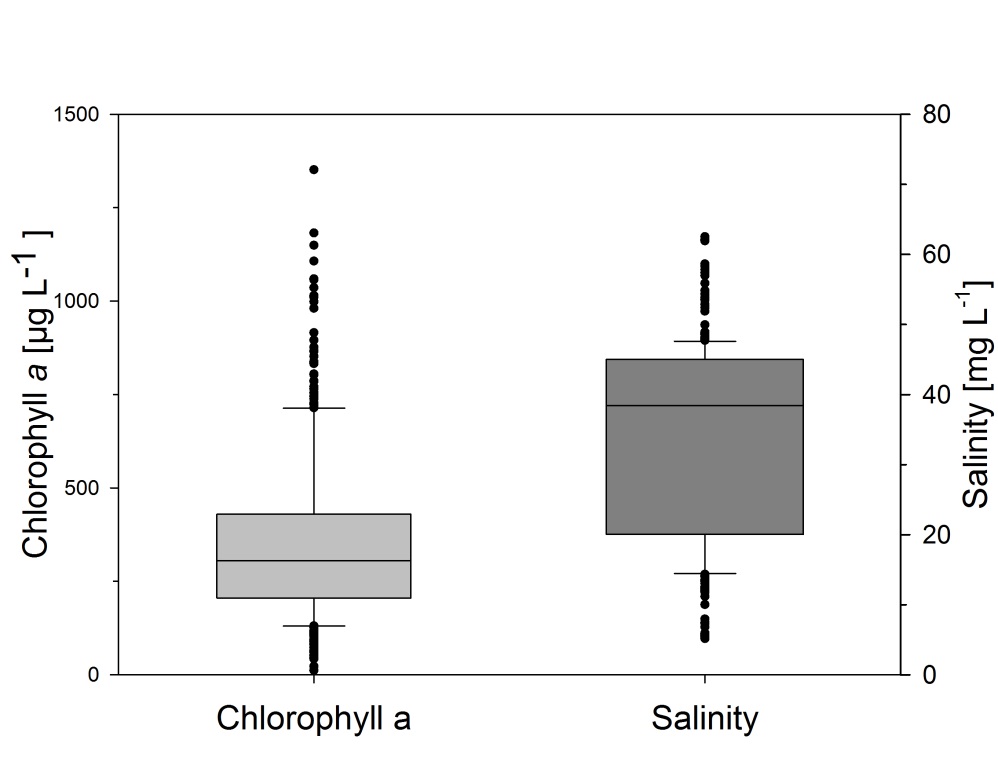


**Figure S1** Salinity and chlorophyll-*a* values from 7 different African soda-lakes (*n* = 412) included in the meta-analysis of rotifer blooms. Boxplots show the median (central line), first and third quartile (box limits), 95% interval and outliers (dots).

**Table S3** Mean values (±standard deviation) and ranges of environmental and biotic variables in Lake Nakuru between January and July 2009.

|  | Mean | | max | min |
| --- | --- | --- | --- | --- |
| Temperature [°C] | 24.9 | ±2.0 | 29.1 | 21.6 |
| Conductivity [mS cm^-1^] | 42.5 | ±7.0 | 55.0 | 30.7 |
| pH | 10.08 | ±0.09 | 10.24 | 9.97 |
| Dissolved organic carbon [mg L^-1^] | 339 | ±68 | 475 | 227 |
| Dissolved nitrogen [mg L^-1^] | 23.4 | ±4.3 | 30.9 | 15.2 |
| Soluble reactive phosphorus [µg L^-1^] | 771 | ±568 | 2035 | 74 |
| Secchi depth [cm] | 23.6 | ±6.1 | 38.0 | 15.0 |
| Particulate matter [mg C L^-1^] | 127.5 | ±49.4 | 247.1 | 36.0 |
| Organic content of particulate matter [%] | 39.7 | ±13.6 | 71.2 | 16.2 |
| Bacterial abundance [ind L^-1^] | 1.8 x 10^8^ | ±6.6 x 10^7^ | 3.1 x 10^8^ | 4.7 x 10^7^ |
| Chl-a [µg L^-1^] | 385 | ±155 | 765 | 197 |
| Total algae biomass [mg C L^-1^] | 19.4 | ±9.0 | 48.6 | 7.3 |

**Figure S2** The relative abundance of filamentous cyanobacteria before and during the onset of rotifer blooms, revealed by the meta-analysis of African soda-lakes. A one-sided paired t-test revealed a significant difference (*p* = 0.028, *n* = 23). In half of the data points, algae communities were already dominated (>80%) before the onsets of blooms. When these data points were excluded a highly significant increase was revealed (*p* < 0.01, *n* = 12).


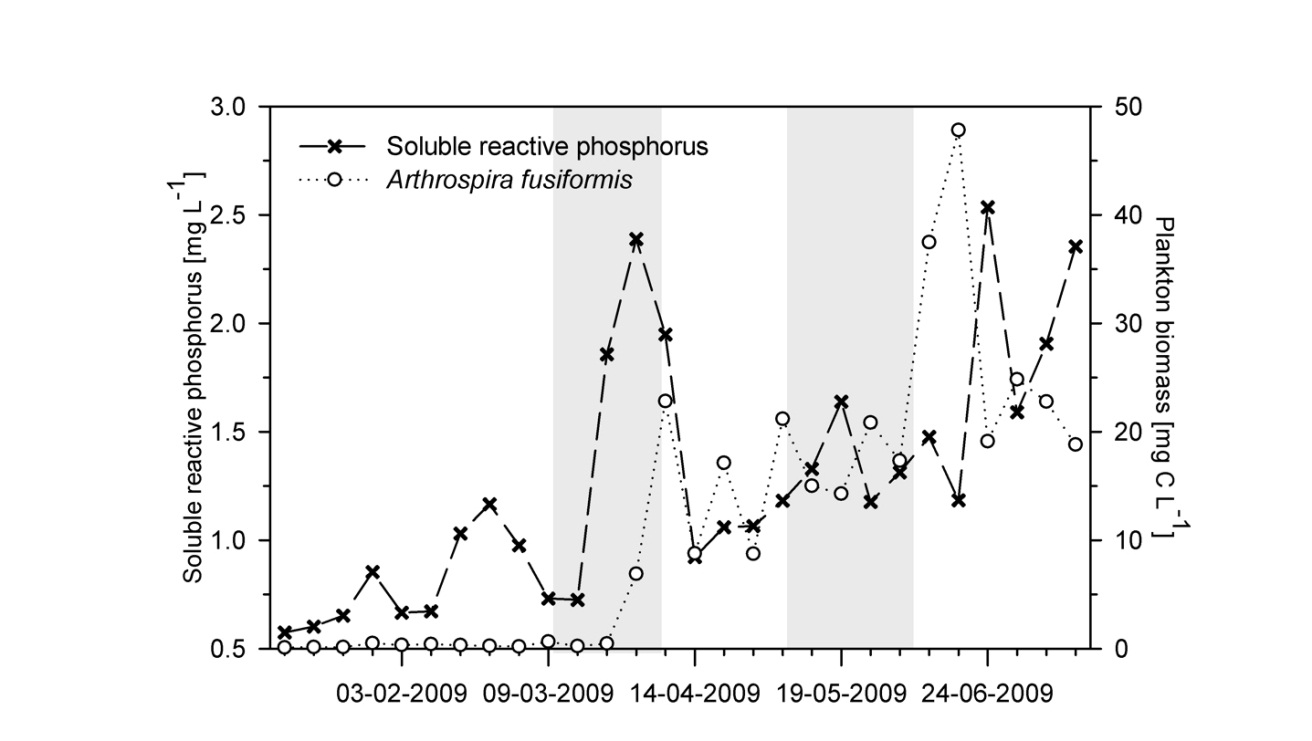


**Figure S3** Trends of soluble reactive phosphorus (SRP), and the biomass of the filamentous cyanobacteria *A. fusiformis* in Lake Nakuru from January to July 2009. Times of rotifer blooms are indicated by grey bars.


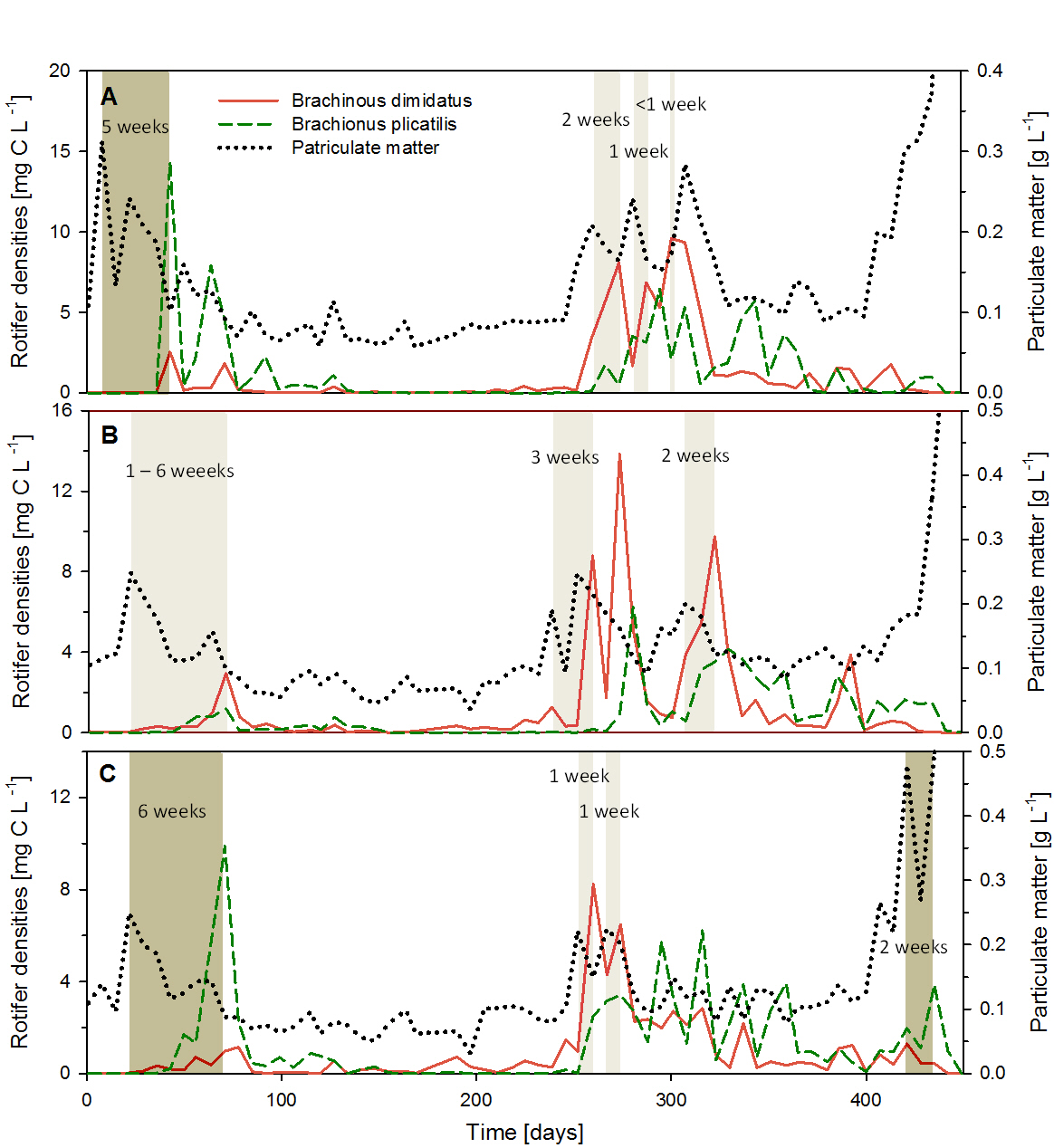


**Figure S4** Temporal dynamics of *Brachionus* species and particulate matter in Lake Nakuru at a southern (A), central (B), and northern (C) sampling station from July 2008 to October 2009. Lag times between particulate matter and rotifer species are indicated in the figure, where light grey bars represent latent periods of *B. dimidiatus* and brown bars indicate latent times of *B. plicatilis*. Increase of particulate matter after day 400 was caused by rapid decrease of lake levels and the desiccation of the lake.

**References**

Ahlgren, G. 1983. Comparison of methods for estimation of phytoplankton carbon. Arch Hydrobiol **98:** 489–508.

Bell, R. T. 1993. Estimating production of heterotrophic bacterioplankton via incorporation of tritiated thymidine. Handb. Aquat. Microb. Meth., p. 495-503.

Menden-Deuer, S., and E. J. Lessard. 2000. Carbon to volume relationships for dinoflagellates, diatoms, and other protist plankton. Limnol Oceanogr **45:** 569-579.

Schroder, T., S. Howard, M. L. Arroyo, and E. J. Walsh. 2007. Sexual reproduction and diapause of Hexarthra sp (Rotifera) in short-lived ponds in the Chihuahuan Desert. Freshwater Biol **52:** 1033-1042.

Strathmann, R. R. 1967. Estimating Organic Carbon Content of Phytoplankton from Cell Volume or Plasma Volume. Limnol Oceanogr **12:** 411-418.

Telesh, I. V., M. Rahkola, and M. Viljanen. 1998. Carbon content of some freshwater rotifers. Hydrobiologia **387:** 355-360.

Vareschi, E., and J. Jacobs. 1984. The Ecology of Lake Nakuru (Kenya). 5. Production and Consumption of Consumer Organisms. Oecologia **61:** 83-98.
